# Lessons from the Khanom nuclear power plant conflict: community resistance, discursive strategies, and sustainable development in southern Thailand

**DOI:** 10.3389/fsoc.2026.1753371

**Published:** 2026-02-26

**Authors:** Kanokkarn Mueangkaew, Sakunrat Yeesakun, Tashi Wangmoc

**Affiliations:** 1 School of Languages and General Education, Center of Excellence in Women and Social Security, Walailak University, Nakhon Si Thammarat, Thailand; 2 Royal Thimphu College, Royal University of Bhutan, Thimphu, Bhutan

**Keywords:** community resistance, discursive strategies, nuclear power, sustainable development, Thailand

## Abstract

**Introduction:**

This study examines community resistance to a proposed nuclear power plant in Khanom District, southern Thailand, as a form of discursive and strategic contestation against hegemonic, state-led development paradigms. It explores how local actors perceived the project and how collective resistance reshaped local understandings of sustainability, development, and community autonomy.

**Methods:**

The study adopts a qualitative research design with phenomenological sensitivity and a participatory orientation to examine lived experiences and meaning-making processes within the resistance movement. Fieldwork was conducted between January and March 2023 and involved 20 in-depth interviews with community leaders, local university graduates, fisherfolk and agricultural residents, local government officers, and civil society actors, selected through purposive and snowball sampling. Data were collected through semi-structured interviews, participant observation, and document analysis, and were analyzed thematically to identify patterns of perception, discourse, and collective action.

**Results:**

Findings reveal that the proposed nuclear power plant was widely perceived as a multidimensional threat to environmental integrity, local livelihoods, and community self-determination. Resistance was galvanized by concerns over the absence of mandatory Environmental Impact Assessments (EIAs), limited public participation, and opaque state decision-making processes. Local leaders and university graduates emerged as key knowledge brokers, translating technical risk information into culturally grounded narratives that mobilized ethical, environmental, and social justice frames. Through this process, sustainability was rearticulated as a locally embedded moral and political claim rather than a technocratic policy objective imposed from above. The conflict revealed persistent structural tensions between centralized national energy governance, represented by the Electricity Generating Authority of Thailand (EGAT), and participatory forms of community-based governance.

**Implications:**

This study contributes to sociological debates on development, social movements, and sustainability by demonstrating how grassroots resistance operates through discursive reconfiguration rather than oppositional protest alone. It highlights the role of knowledge brokerage in shaping collective action and underscores the need for energy governance reforms in Thailand that institutionalize meaningful public participation, ensure transparent and independent EIAs, and recognize community-led alternatives within national energy planning frameworks.

## 1 Introduction

Contemporary development approaches in many developing countries, including Thailand, have long been shaped by state-led modernization paradigms that prioritize economic growth, industrial expansion, and technological advancement as primary indicators of progress. Rooted in Western modernization theory, this paradigm emphasizes integration into global markets and large-scale infrastructure development as central strategies for national advancement (Rostow, 1960; Phongpaichit and Baker, 2009). In the Thai context, modernization has historically been pursued through a centralized development model in which state agencies and state-linked corporations play a dominant role in planning and implementation, often marginalizing local participation and place-based knowledge. Partnerships between government institutions and domestic or transnational corporations have formed what scholars describe as a “growth coalition,” driving resource-intensive projects framed as national necessities rather than subjects of democratic deliberation (Yang et al., 2015).

Critical development scholarship has increasingly questioned the presumed neutrality of such modernization trajectories, highlighting how technocratic decision-making and growth-oriented policies obscure social inequalities, environmental costs, and democratic deficits (Escobar, 1995; Pieterse, 2000). In Thailand, government-backed development projects have frequently encroached upon community lands, forests, water resources, and coastal ecosystems, fundamentally altering traditional systems of resource management and local livelihoods (Walker, 2001; Lunchaprasith, 2017). The environmental consequences of these projects including deforestation, pollution, biodiversity loss, and ecological degradation extend beyond ecological damage to threaten human well-being, health, and cultural practices, particularly among communities dependent on agriculture, fishing, and natural resources (Rigg and Salamanca, 2011; Phongpaichit and Baker, 2017).

These tensions become particularly pronounced in the governance of high-risk energy technologies such as nuclear power. Unlike other contested infrastructure projects, nuclear development collapses the boundary between technical expertise and political authority, allowing state actors to legitimize exclusionary decision-making through claims of scientific necessity, national security, and risk management. This fusion of expert knowledge and sovereign power renders nuclear governance especially resistant to democratic scrutiny and meaningful local participation, thereby intensifying conflicts between centralized state authority and community-based claims to environmental safety and democratic accountability (Shrader-Frechette, 2011; Ramana, 2020). As a result, nuclear energy foregrounds fundamental questions of state legitimacy, moral responsibility, public accountability, and intergenerational justice. Decision-making processes surrounding nuclear projects are frequently characterized by limited transparency, restricted public deliberation, and an overreliance on expert authority conditions that tend to deepen public distrust and amplify community concern (Greacen and Footner, 2001). In this sense, nuclear power constitutes a distinctive site of political contestation rather than merely another form of centralized infrastructure development.

In response to the social and environmental impacts of state-led development, alternative paradigms emphasizing sustainability, participation, and community agency have gained prominence among scholars, civil society organizations, and affected communities. Contemporary sustainable development frameworks conceptualize development as a multidimensional process that integrates economic viability, environmental protection, and social justice, with an emphasis on preserving resources for future generations (Mensah, 2019). Central to these approaches is community participation, which recognizes local knowledge, cultural values, and lived experience as essential components of legitimate and effective development planning (Chambers, 1997; Cornwall, 2003). Such perspectives challenge top-down development models that impose externally defined priorities while neglecting local contexts and uneven distributions of risk and benefit an issue that becomes especially salient in high-risk energy governance.

Unlike other large-scale infrastructure projects commonly contested in Thailand such as dams, industrial estates, or coal-fired power plants nuclear energy occupies a qualitatively distinct position in the politics of development. In this study, discourse is understood not merely as language but as a practical mechanism through which authority, legitimacy, and acceptable knowledge are constructed and contested in everyday governance encounters. Nuclear projects are characterized by irreversibility, long temporal horizons of risk, and epistemic closure through expert-dominated governance. The risks associated with nuclear power exceed the scale of local mitigation or compensation and extend beyond the lifespan of existing institutions. In the Thai context, where energy governance is centralized within the Electricity Generating Authority of Thailand (EGAT) and public participation mechanisms remain procedurally limited, nuclear development intensifies legitimacy deficits by concentrating authority, restricting deliberation, and depoliticizing risk through technical discourse. As such, resistance to nuclear power in Khanom cannot be understood as a generic infrastructure conflict, but as a confrontation with a development model that collapses democratic decision-making into technocratic necessity.

Thailand has witnessed numerous social movements contesting mainstream development projects, particularly those perceived as threatening natural resources, livelihoods, and community autonomy. These movements have employed a range of strategies, including peaceful protest, legal action, public campaigns, and, in some cases, direct confrontation with state authorities (Forsyth, 2014; Carè and De Lisa, 2019). Social movement theory drawing on concepts such as resource mobilization, political opportunity structures, and framing processes offers valuable insights into how communities organize collective action, mobilize support, and challenge dominant development paradigms (Zurcher and Snow, 2017). However, existing studies often emphasize organizational dynamics and political outcomes, paying comparatively less attention to how resistance is grounded in everyday lived experience and the moral meanings communities attach to development, risk, and sustainability.

Nuclear power development in Thailand has generated particularly strong opposition from local communities, environmental groups, and civil society organizations concerned with safety, ecological protection, and democratic participation in energy planning. Proposed nuclear power plants have been met with resistance strategies that include scientific risk assessments, legal challenges, alternative energy proposals, and public education campaigns, often involving collaboration among local residents, academics, technical experts, and non-governmental organizations (Glassman, 2010; Nilaphatama, 2020; Kooyai and Jitpiromsri, 2024). These conflicts reveal not only struggles over specific projects but also broader debates about the meaning of development, sustainability, and the role of communities in shaping national energy futures.

In Thailand, nuclear energy planning has historically been centralized within state technocratic institutions, most notably EGAT, with limited institutional mechanisms for substantive public deliberation or community consent. Although nuclear power has not been implemented, recurring policy proposals, feasibility studies, and national energy plans have repeatedly revived public concern. These cycles of proposal and suspension reveal a persistent governance gap between centralized energy planning and local democratic accountability, rendering nuclear power a symbolic and political focal point for broader struggles over participation, transparency, and development legitimacy.

The case of Khanom District in Nakhon Si Thammarat Province provides a compelling illustration of these dynamics. Characterized by rich coastal, forest, and agricultural ecosystems, Khanom supports livelihoods based on fishing, farming, and tourism. The proposed nuclear power plant posed perceived threats to environmental integrity, public health, and the sustainability of local livelihoods, prompting sustained resistance from community members (Greenpeace Southeast Asia, 2014; Hirsch, 2010). Beyond organized protest, this resistance was rooted in residents’ lived experiences of environmental vulnerability, livelihood insecurity, and perceived exclusion from decision-making processes. By foregrounding these experiences, the study adopts a phenomenological orientation that treats resistance not only as political action but as an embodied and meaning-making process through which communities interpret, feel, and morally evaluate development and risk.

Despite a growing body of research on anti-nuclear movements and infrastructure conflicts in Asia, existing studies have largely emphasized organizational strategies, policy outcomes, and expert-driven risk debates. Far less attention has been paid to how community resistance is grounded in lived experience and how phenomenological meanings are translated into discursive practices that contest dominant sustainability narratives particularly in Southeast Asian governance contexts characterized by centralized authority and limited participatory mechanisms. As a result, the micro-level processes through which communities reconstitute development, risk, and legitimacy from below remain underexplored.

This article contributes to sociological scholarship by advancing three interrelated arguments. First, it reconceptualizes community resistance not merely as oppositional action but as a process of discursive reconfiguration through which local actors actively redefine the meaning of development itself. Second, it highlights the role of local university graduates as discursive intermediaries who translate technical knowledge into culturally resonant narratives grounded in lived experience, thereby enabling grassroots mobilization and collective meaning-making in the Global South. Third, it reframes sustainability as a contested moral discourse mobilized from below rather than as a technocratic policy objective imposed from above. In doing so, the article moves beyond descriptive accounts of resistance by offering a theoretically grounded explanation of how phenomenological experience, discursive strategy, and power relations intersect in contested energy governance in the Global South.

## 2 Research questions

To guide the inquiry and ensure an analytically grounded examination of community resistance, this study is structured around the following research questions:

- How do community members in Khanom District experience, interpret, and discursively construct the proposed nuclear power plant in relation to perceived risk, livelihood security, and everyday sustainability?
- Through which social actors, knowledge practices, and discursive strategies does local resistance emerge, circulate, and become institutionalized over time?
- How does the resistance movement generate alternative sustainability imaginaries that contest and reconfigure dominant state-led development paradigms in Thailand?

**Figure 1:**
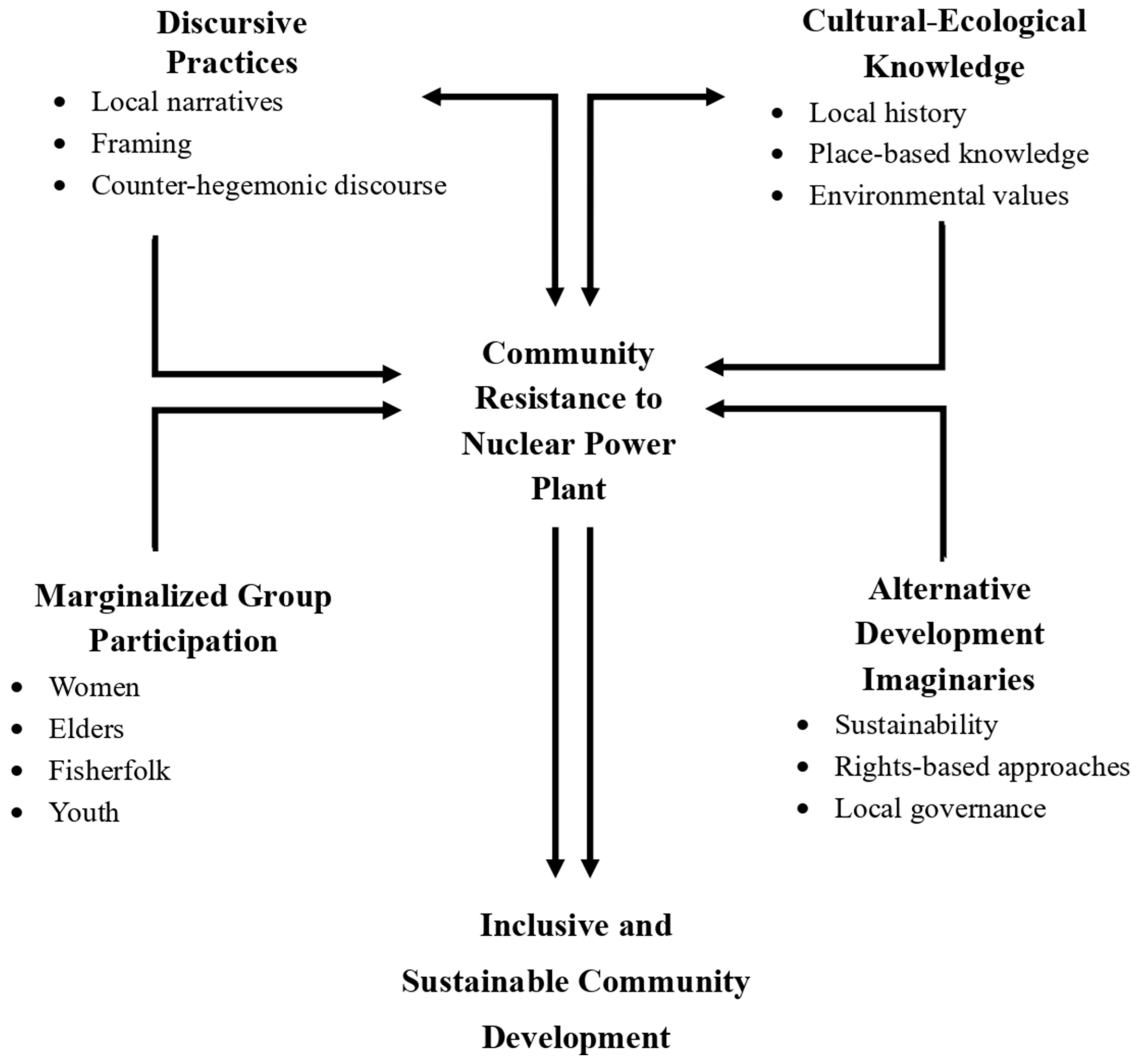
Conceptual framework of community resistance to nuclear power plant development.

These questions are significant as they provide a foundation for understanding not only resistance to externally imposed development models but also how such resistance can serve as a site of local knowledge production. In a broader sense, these questions contribute to critical development studies by highlighting the importance of community agency, cultural sustainability, and participatory alternatives in the Global South in Figure 1.

The arrows in this framework indicate analytical relationships rather than deterministic causal pathways, reflecting the dynamic, relational, and non-linear nature of discursive struggle observed in the field.

Figure 1 presents the analytical framework guiding this study’s examination of community resistance to the proposed nuclear power plant in Khanom District. The framework conceptualizes resistance as an emergent and relational process arising from the interaction of four interrelated dimensions: discursive practices, cultural ecological knowledge, marginalized group participation, and alternative development imaginaries. These dimensions are not treated as linear stages or independent causal variables, but as mutually constitutive analytical processes that together shape collective action and meaning-making.

- Discursive practices refer to the framing strategies, narratives, and counter-hegemonic discourses through which community actors articulate critiques of nuclear development and state-led modernization. Drawing on discourse and framing theory, this dimension captures how concepts such as risk, sustainability, and development are actively redefined in opposition to technocratic and growth-oriented narratives promoted by state institutions (Dryzek, 2013). Discourse, in this sense, operates as a site where power, legitimacy, and authority are negotiated rather than imposed.
- Cultural ecological knowledge encompasses place-based environmental understandings, historical experiences of resource governance, and locally embedded ecological values that inform community interpretations of nuclear risk. This dimension highlights how resistance is grounded in lived relationships with coastal, agricultural, and marine ecosystems, rather than abstract technical assessments of safety (Escobar, 1995). Such knowledge provides the moral and experiential foundation upon which resistance discourses gain legitimacy and resonance within the community.
- Marginalized group participation foregrounds the involvement of social actors whose voices are often excluded from formal decision-making processes, including women, fishers, elders, and youth. This dimension emphasizes how everyday experiences of livelihood insecurity, environmental vulnerability, and social marginalization become central resources for resistance mobilization (Cornwall, 2003). Participation by these groups anchors resistance in daily life and challenges elite-dominated representations of development, expertise, and risk.
- Alternative development imaginaries capture the collectively articulated visions of sustainability, rights-based development, and local governance that emerge through resistance practices. Rather than rejecting development outright, community actors advance alternative imaginaries emphasizing environmental stewardship, food security, intergenerational responsibility, and democratic participation. This dimension aligns with critical development scholarship that conceptualizes development as a contested moral and political project rather than a neutral policy objective (Sachs, 2015).

At the center of the framework, community resistance is understood as an emergent process produced through the dynamic interaction of these four dimensions. The arrows in Figure 1 represent ongoing analytical relationships, indicating how discursive practices, knowledge systems, participation, and imaginaries continuously shape and reinforce one another over time. Resistance is thus conceptualized not as a fixed outcome, but as an ongoing process of negotiation, reinterpretation, and discursive reconfiguration. The framework was developed inductively from empirical analysis, while being theoretically informed by social movement theory, discourse analysis, and critical development studies. By integrating lived experience, discourse, and power relations, the framework enables a nuanced sociological analysis of how communities contest nuclear development and actively redefine sustainability from below.

## 3 Literature review

### 3.1 Development discourse and the politics of expertise

Development discourse has evolved alongside global political–economic transformations. Early modernization theory framed development as a linear transition from “traditional” to “modern” societies based on Western industrial trajectories (Rostow, 1960). This paradigm has been widely criticized for its ethnocentrism, technocratic bias, and marginalization of local knowledge (Escobar, 1995; Grillo, 2020). Dependency and post-development perspectives further revealed how development projects often reproduce inequality by embedding peripheral regions into extractive and unequal power relations (Frank, 1967; Pieterse, 2000).

Foucauldian approaches reconceptualize development as a mode of governance enacted through expertise, knowledge production, and disciplinary power (Foucault, 1977, 1980). Large-scale infrastructure projects particularly nuclear energy operates through expert rationalities that depoliticize political decisions and legitimize exclusionary practices. Scientific authority and risk discourse often function to marginalize community concerns and constrain democratic deliberation (Scott, 2023; Shrader-Frechette, 2011). This study builds on these insights by examining nuclear development in Thailand as a site where technocratic authority and community-based knowledge come into direct conflict.

### 3.2 Social movements, discourse, and energy democracy

Classical social movement theories emphasize resources, political opportunities, and organizational capacity (Zurcher and Snow, 2017). While analytically useful, these approaches often underplay the discursive and moral dimensions through which movements construct meaning and legitimacy. More recent scholarship foregrounds framing processes, identity, and everyday experience as central to collective mobilization (Gillan, 2019; Della Porta, 2020a).

In energy conflicts, these dynamics are increasingly analyzed through the concept of energy democracy, which critiques centralized energy governance and advocates participatory, decentralized, and socially accountable energy systems (Burke and Stephens, 2017). Anti-nuclear movements in Asia illustrate this shift. In India, resistance to the Kudankulam nuclear project emphasized democratic consent and epistemic injustice in expert-led risk assessments (Kaur, 2018). In Taiwan, anti-nuclear activism linked energy policy to democratization and environmental citizenship (Ho, 2014), while South Korean movements highlighted transparency and civic participation in nuclear governance (Kim, 2019). These cases demonstrate that anti-nuclear resistance functions not only as opposition but also as a broader critique of technocratic development.

### 3.3 Sustainable community development as a contested practice

Sustainable community development (SCD) extends beyond the Brundtland framework by emphasizing participation, equity, and local governance (World Commission on Environment and Development, 1987). Participatory development scholars argue that sustainability requires meaningful community involvement rather than top-down planning (Chambers, 1997; Cornwall, 2003). Feminist and critical perspectives further highlight how sustainability is shaped by gendered and unequal access to resources (Kabeer, 1999). At the same time, sustainability can be co-opted as a technocratic discourse to legitimize environmentally risky projects under the banner of “clean energy” (Sachs, 2015). Nuclear power is frequently framed as a low-carbon solution while sidelining local concerns about long-term ecological risk and democratic accountability. This study contributes to SCD debates by conceptualizing sustainability as a moral and political discourse articulated from below.

### 3.4 Community resistance to nuclear development: comparative perspectives

Studies of anti-nuclear movements consistently highlight distrust toward expert authority, contested risk knowledge, and struggles over democratic participation. Research in Europe, the United States, and Japan demonstrates how transparency deficits and historical accidents have shaped resistance (Johnston, 2010; Darr, 2020). In Asia, anti-nuclear activism increasingly links local opposition to broader demands for democratic governance and alternative energy futures (Ho, 2014; Kim, 2019; Kaur, 2018). In Thailand, research on community resistance has focused mainly on mining, industrial pollution, and dams (Walker, 2012), with limited attention to nuclear energy as a discursive arena. This study addresses this gap by examining the Khanom anti-nuclear movement as a case of grassroots resistance that challenges hegemonic development narratives and advances alternative visions of sustainable community development grounded in lived experience.

## 4 Research design and methodology

This study adopts a qualitative research design to examine how community resistance to the proposed nuclear power plant in Khanom District is constructed, experienced, and sustained through everyday practices, meanings, and discourses. Resistance is approached not as a fixed outcome but as a socially produced process shaped by lived experience, local knowledge, and power relations. Methodologically, the study is informed by phenomenological sensibilities, particularly its attention to participants’ meaning-making, without advancing a strong transcendental phenomenological claim. This positioning allows the analysis to foreground experiential perspectives while situating them within broader socio-political and discursive contexts (Finlay, 2012; van Manen, 2016). The approach is therefore interpretive and inductive, informed by development studies and social movement theory.

### 4.1 Fieldwork period and participants

Fieldwork was conducted between January and March 2023 in Khanom District, Nakhon Si Thammarat Province. While the analysis was finalized in 2026, the identified discursive structures and phenomenological essences particularly regarding state-community power dynamics and ontological security remain highly salient within Thailand’s contemporary socio-political landscape of nuclear energy. The findings reflect enduring structural tensions rather than transient sentiments. The study involved 20 in-depth interviews with community leaders, local university graduates, fisherfolk and agricultural residents, local government officers, and civil society actors. Participants were selected through purposive and snowball sampling, beginning with individuals directly involved in community mobilization or local development processes. Snowball sampling enabled access to informal leaders, women, elders, and retired activists, ensuring diversity in gender, age, occupation, and social position. This sampling strategy captured varied interpretations of nuclear development, risk, and sustainability.

### 4.2 Data collection

Data were generated through semi-structured interviews, participant observation, and document analysis. Interviews focused on experiences of resistance, perceptions of nuclear risk, state community relations, and visions of sustainable development. Participant observation was conducted during community meetings and informal gatherings, while documentary sources including policy documents, NGO materials, media reports, and community records were used to contextualize interview data (Table 1).

**Table 1:** Table of interviewees.

ID	Gender	Age range	Primary occupation	Role/perspective in the movement
P01	Male	50–60	Small-scale Fisher	Community Leader & Oral Historian
P02	Female	40–50	Small-scale Fisher	Women’s Group Representative
P03	Male	30–40	Fisher/Local Trader	Logistics & Community Mobilizer
P04	Male	60+	Retired Teacher	Advisor on Local Rights & Policy
P05	Female	20–30	University Graduate	Digital Activist & Information Broker
P06	Male	20–30	University Graduate	Youth Coordinator & Researcher
P07	Male	40–50	Fruit Orchard Farmer	Land Rights Advocate
P08	Female	50–60	Agriculturalist	Community Welfare Organizer
P09	Male	40–50	Local Govt. Officer	Information Provider (Institutional context)
P10	Female	30–40	Local Govt. Officer	Public Relations & Conflict Mediator
P11	Male	50–60	Civil Society Actor	Regional Network Coordinator
P12	Female	40–50	Civil Society Actor	Environmental Education Specialist
P13	Male	30–40	Small-scale Fisher	Marine Resource Monitoring
P14	Female	20–30	University Graduate	Communication & Graphic Support
P15	Male	60+	Religious Leader	Moral Authority & Peaceful Protest Lead
P16	Male	40–50	Local Business Owner	Resource Provider & Financial Supporter
P17	Female	30–40	Fisher/Housewife	Grassroots Mobilizer
P18	Male	50–60	Rubber Farmer	Area Liaison & Security Volunteer
P19	Female	40–50	Local Health Worker	Risk Perception & Health Advocate
P20	Male	30–40	Civil Society Actor	Legal Aid & Documentation

Following phenomenological reduction, the analysis moved beyond surface narratives to identify invariant meaning structures that recurred across participants’ accounts, particularly regarding perceived irreversibility, intergenerational risk, and moral responsibility associated with nuclear development.

### 4.3 Data analysis

The data analysis was informed by Husserlian phenomenological principles (Husserl, 1997), rather than a strict transcendental phenomenological procedure, specifically utilizing the following four-stage coding workflow to ensure phenomenological reduction (Epoché):

- Bracketing and Immersion (Epoché): Researchers practiced bracketing by setting aside prior assumptions about nuclear energy and state-led development. We began by repeatedly reading the transcripts to achieve a holistic sense of the participants lived experiences regarding the Khanom conflict.
- Phenomenological Reduction (Extracting Meaning Units): The text was broken down into meaning unit’s specific statements that capture the essence of how participants perceive risks, the state, and their environment. We looked beyond the facts to find the noema (what is experienced) and noesis (how it is experienced). These concepts were used heuristically to guide interpretation, rather than as formal philosophical abstractions.
- Thematic Clustering: These meaning units were clustered into broader themes. For instance, individual expressions of fear of the unknown and distrust in technical experts were synthesized into the theme of Erosion of Ontological Security.
- Synthesis of Essence: Finally, we integrated the transformed themes into a consistent statement of the essential structure of the phenomenon identifying the core discursive strategies and the alternative sustainability imaginaries constructed by the community.

While Phenomenology captures the internal meaning-making and lived experiences of the residents, Discourse Analysis allows us to see how these internal meanings are externalized as strategic repertoires to challenge state power. Thus, we use Phenomenology to understand the source of resistance and Discourse Analysis to understand its application (Hajer, 1995; Fairclough, 2013).

Power is operationalized in this study not as coercive force, but as the capacity to define legitimate knowledge, acceptable risk, and authoritative narratives of development. Following Foucault (2020) perspectives, discourse is traced empirically through recurring patterns in how state actors frame nuclear energy as technical necessity, how participation is procedurally constrained, and how community knowledge is delegitimized as emotional or unscientific.

Conversely, resistance is analyzed as counter-discursive practice through which community actors reassert epistemic authority by grounding sustainability in lived experience, moral responsibility, and ecological relations. Power thus becomes observable in moments of discursive exclusion, translation, and redefinition rather than formal institutional outcomes alone.

### 4.4 Trustworthiness and triangulation

To enhance trustworthiness, the study employed methodological and investigator triangulation (Denzin, 2006). Interview data were cross-checked with observations and documents, and perspectives from community members were compared with those of local officials. Three assistant researchers participated in preliminary coding and thematic discussions to reduce individual bias. This multi-source and multi-analyst approach strengthens the credibility and analytical rigor of the findings.

## 5 Results

### 5.1 Structures and chronology of the anti-nuclear protest movement in Khanom District, Thailand

This section traces the temporal development and structural dynamics of the anti-nuclear protest movement in Khanom District, highlighting key turning points through which local opposition emerged, intensified, and became organized collective resistance. Rather than presenting resistance as a spontaneous reaction, the findings demonstrate how opposition unfolded through a sequence of events shaped by governance practices, information flows, and lived experience.

#### 5.1.1 Background: nuclear energy planning and the re-emergence of Khanom as a proposed sit

Thailand’s engagement with nuclear power dates back to 1966, when the Electricity Generating Authority of Thailand (EGAT) first proposed a nuclear power plant at Ao Phai, Chonburi Province. Although feasibility studies were approved and uranium fuel ordered, the project was indefinitely postponed in 1978 following the discovery of natural gas reserves in the Gulf of Thailand. Nuclear planning resurfaced four decades later with the approval of the Power Development Plan 2007 (Electricity Generating Authority of Thailand, 2007) by the National Energy Policy Council (NEPC). The plan envisioned nuclear power plants becoming operational by 2020–2021 and allocated funding for preparatory infrastructure between 2008 and 2010. Khanom District in Nakhon Si Thammarat Province was identified as one of several potential sites, alongside locations in Surat Thani, Trat, and Nakhon Sawan. Although nuclear power was never implemented, repeated feasibility studies and site surveys placed Khanom under sustained policy attention, creating prolonged uncertainty among residents. This uncertainty formed the structural backdrop against which community resistance later emerged.

#### 5.1.2 Turning point I: absence of EIA and the breakdown of trust (2008–2010)

The first major turning point occurred when EGAT initiated geological surveys in Khanom without conducting a mandatory Environmental Impact Assessment (EIA) or formally informing local communities. While EGAT commissioned Burns & Roe (USA) to identify potential sites nationwide, no site-specific EIA was undertaken for Khanom. Survey activities were carried out quietly, triggering suspicion and anxiety among villagers. Residents interpreted the absence of an EIA not merely as a technical omission, but as evidence of institutional disregard for local participation and environmental governance. Participants consistently emphasized that nuclear power was qualitatively different from other large-scale infrastructure projects, describing it as irreversible, transgenerational, and beyond the scope of financial compensation or conventional mitigation measures. One fisherman recalled:

*“We grew up with the sound of the waves and the scent of the sea. For us, the ocean is not merely a place to earn a living; it is our very life. When they announced the plan for the nuclear power plant, my first reaction wasn’t just fear of an explosion. It was the feeling of being evicted from my own home with nowhere else to go. They [state officials] arrived with a single piece of paper claiming it was ‘safe,’ yet they never bothered to step onto our boats. They never saw how our hands are stained with mud when we harvest clams. This attempt by the state to impose a version of ‘progress’ that we never asked for makes us feel as though our voices are so faint that they can simply pretend not to hear us.” (P01).*

This narrative illustrates the ontological insecurity felt by the local community, where the technocratic safety promised by the state fails to acknowledge the lifeworld and deep-rooted connection between the residents and their environment.

#### 5.1.3 Turning point II: knowledge mobilization and the emergence of collective awareness

Initially, many villagers expressed indifference or resignation, viewing nuclear power as a distant, technical issue beyond local control. Resistance began to crystallize when local leaders and university graduates many of whom had returned to Khanom started researching nuclear risks and translating technical information into accessible language. Community meetings were organized, informal discussions were held in fishing docks and village houses, and visual protest signs began appearing in residential areas. A senior community member reflected:

*“At first, we did not understand what a nuclear plant really meant. But when the young people explained the dangers, we realized it was not just electricity it was about our sea, our food, and our children’s future.” (P16).*

This phase illustrates how knowledge brokerage functioned as a catalyst for resistance, converting individual concern into shared moral and political understanding.

#### 5.1.4 Turning point III: expansion of the movement and networked support

As awareness of nuclear risks deepened, the resistance movement expanded beyond its initial core of community leaders to encompass a broader constellation of actors, including fisherfolk, agricultural residents, women’s groups, elders, local environmental engineers, university-based researchers, NGO activists, and media practitioners. This expansion marked a shift from localized concern to networked collective action, as external expertise and advocacy resources were selectively incorporated to strengthen the movement’s legitimacy and visibility.

University graduates played a particularly significant role as knowledge brokers, translating technical reports and policy language into accessible narratives grounded in local experience. A young graduate involved in information dissemination explained:

*“If we talked only in technical terms, people would feel it had nothing to do with them. We had to explain nuclear power through everyday life what happens to fish, to water, to children growing up here.” (P05).*

Through these translation practices, abstract risk assessments were rearticulated as concrete threats to livelihood security, food systems, and intergenerational responsibility. Women participants similarly emphasized how nuclear risk intersected with household and caregiving concerns. One fisherwoman noted:

“When officials talked about electricity, I thought about cooking, about feeding my family, about whether our seafood would still be safe. That is why I could not stay silent.” *(P02).*

In response to growing opposition, EGAT intensified Corporate Social Responsibility (CSR) initiatives, including funding for schools, infrastructure repairs, and community donations. While framed by the state as gestures of goodwill, many residents interpreted these actions as attempts to neutralize dissent without addressing substantive concerns about safety, participation, and long-term environmental risk. A senior community organizer remarked:

*“They offered money for buildings, but they never answered our questions. It felt like they wanted to buy silence, not trust.” (P11).*

Rather than pacifying resistance, these strategies reinforced perceptions of asymmetrical power and deepened skepticism toward state intentions, further consolidating collective opposition.

#### 5.1.5 Power structures and discursive conflict

The unfolding conflict revealed entrenched power asymmetries between centralized state institutions most notably EGAT and local communities. State actors consistently framed nuclear energy as a low-cost, environmentally friendly solution necessary for national energy security, relying on technical expertise and future-oriented economic rationales to legitimize decision-making. In contrast, community narratives foregrounded environmental vulnerability, livelihood precarity, and democratic exclusion, challenging the authority of technocratic risk discourse.

Local administrative organizations occupied an ambiguous intermediary position. While some officials expressed personal sympathy toward community concerns, their institutional roles limited their capacity to influence national energy planning. As one local government officer acknowledged:

*“We listen to the villagers, but in the end, decisions come from above. Our role is to explain policies, not to decide them.” (P09).*

This discursive imbalance transformed resistance into more than opposition to a specific infrastructure project. It became a broader struggle over who holds the authority to define legitimate knowledge, acceptable risk, and the meaning of sustainable development. Nuclear risk, initially presented as a calculable technical probability, was redefined by community actors as an existential and moral issue rooted in lived experience and place-based ecological relations.

Through this process, power became visible not primarily through coercion or legal enforcement, but through discursive practices that included exclusion from meaningful deliberation, selective recognition of expertise, and competing claims to moral legitimacy. Resistance in Khanom thus functioned as an ongoing contestation over development itself, challenging the reduction of democratic decision-making to technocratic necessity and asserting alternative sustainability imaginaries articulated from below.

### 5.2 Power structures and discursive influence in the Khanom nuclear power plant conflict

The conflict surrounding the proposed nuclear power plant in Khanom District reveals a structured asymmetry of power between centralized state institutions and local communities, mediated through competing discourses of development, risk, and sustainability. At the institutional center of this configuration is the Electricity Generating Authority of Thailand (EGAT), which operates within a highly centralized governance framework that concentrates authority over energy planning, site selection, and risk assessment. This institutional arrangement substantially constrained opportunities for meaningful community participation and deliberative engagement. A community participant described this imbalance during a public hearing:

*“During the public hearing, they spoke about safety levels and wind directions using complex statistical models. But for us, the wind isn’t just a variable on a graph. We know that in Khanom, the wind carries the salt that nourishes our orchards and the currents that bring the fish to our nets. When we tried to explain how a nuclear plant would disrupt this delicate balance, they looked at us as if we were speaking an extinct language.” (P04).*

This narrative illustrates how technocratic forms of knowledge functioned as a gatekeeping mechanism, privileging expert authority while marginalizing place-based ecological knowledge. By categorizing local experience as “unscientific,” state actors effectively depoliticized the conflict and limited the community’s capacity to participate as legitimate knowledge holders in decisions affecting their environment and livelihoods.

Within this top-down framework, nuclear power was consistently framed by state institutions as a technical and national necessity an efficient, low-carbon solution to long-term energy security. Such framing relied on expert-driven rationality and future-oriented economic projections, positioning local concerns as secondary to national development imperatives and thereby narrowing the scope for democratic scrutiny. Another participant reflected on this discursive encounter:

*“They used words like ‘efficiency’ and ‘strategic location,’ but what we saw were outsiders telling us that the sea our ancestors cared for was somehow inefficient without a power plant. We weren’t just opposing nuclear energy; we were trying to prove that our knowledge matters.” (P03).*

This fragment highlights a clash of epistemologies between technocratic development discourse and localized environmental knowledge. Resistance, in this sense, was not simply opposition to technology but a struggle for recognition, legitimacy, and the authority to define risk and sustainability. In response, the resistance movement articulated a counter-discourse that challenged both the substance and authority of the state’s narrative. A key structural feature of this counter-discourse was the role of local university graduates who returned to Khanom and acted as knowledge brokers. By translating technical information on nuclear risks into locally meaningful terms, these actors bridged expert knowledge and lived experience. Through community meetings, informal discussions, and public forums, villagers particularly fisherfolk and agricultural residents were able to reinterpret the nuclear project not as abstract national infrastructure but as a direct threat to everyday life, health, and livelihood security.

Grassroots strategies further amplified this counter-discourse. Public demonstrations, protest signage, and strategic engagement with local and national media reframed the debate around environmental vulnerability, marine ecosystems, and food security. EGAT’s Corporate Social Responsibility (CSR) initiatives, such as school renovations and material donations, were widely perceived as symbolic gestures that failed to address core concerns regarding risk, transparency, and participation. Rather than mitigating opposition, these initiatives reinforced perceptions of instrumental governance and deepened mistrust.

At the administrative level, the Khanom case exposes a persistent governance gap between national policy objectives and local democratic accountability. While state institutions promoted nuclear energy as a pillar of sustainable development, local actors emphasized inadequate consultation, limited transparency, and long-term ecological uncertainty. This disjuncture demonstrates that sustainability operates not as a shared policy framework but as a contested discourse, structured by unequal power relations and competing claims to knowledge and legitimacy (Table 2).

**Table 2:** Discursive strategies in the Khanom resistance movement.

Discursive frame	Key actors	Target audience	Channels	Intended effect
Nuclear risk as moral threat	Fishers, elders	Local community	Meetings, storytelling	Build moral urgency
Sustainability as food security	Women, vendors	Media, NGOs	Banners, interviews	Reframe development
Expert knowledge translation	University graduates	Villagers	Workshops, social media	Enable understanding
Legitimacy critique	Civil society actors	State agencies	Petitions, hearings	Challenge authority

### 5.3 Discursive struggle, resource mobilization, and developmental impacts

The anti-nuclear protest movement in Khanom constitutes a sustained discursive struggle against Thailand’s dominant development paradigm. Led by community leaders and local university graduates, the movement did not reject development per se but contested the technocratic and growth-centric interpretation embedded in state-led energy planning. Drawing selectively on the language of sustainable development articulated in Thailand’s 9th–11th National Economic and Social Development Plans (2002–2015), protesters reappropriated official rhetoric to advance an alternative, community-centered vision.

#### 5.3.1 Framing resistance through sustainability and food security

Addressing Research Question 1, the movement strategically reframed opposition to nuclear power through sustainability discourse grounded in everyday livelihood concerns. Central to this framing was the notion of food security, which linked ecological integrity to social reproduction and intergenerational responsibility. Protesters emphasized that the proposed site encompassed marine ecosystems supporting artisanal fisheries and endangered species, including the pink dolphin. By foregrounding these ecological relations, the movement repositioned environmental protection as a national concern rather than a parochial interest.

This framing directly challenged the state’s narrative that prioritized centralized energy production while downplaying environmental externalities and long-term social costs. The resulting discursive confrontation reflects a broader tension between technocratic development rationalities and ecological justice frameworks (Escobar, 1995).

This five-dimension model was inductively derived from recurrent empirical patterns in the data and subsequently refined through dialog with Foucauldian perspectives on discourse, power, and resistance.

#### 5.3.2 Resource mobilization and collective action

**Figure 2:**
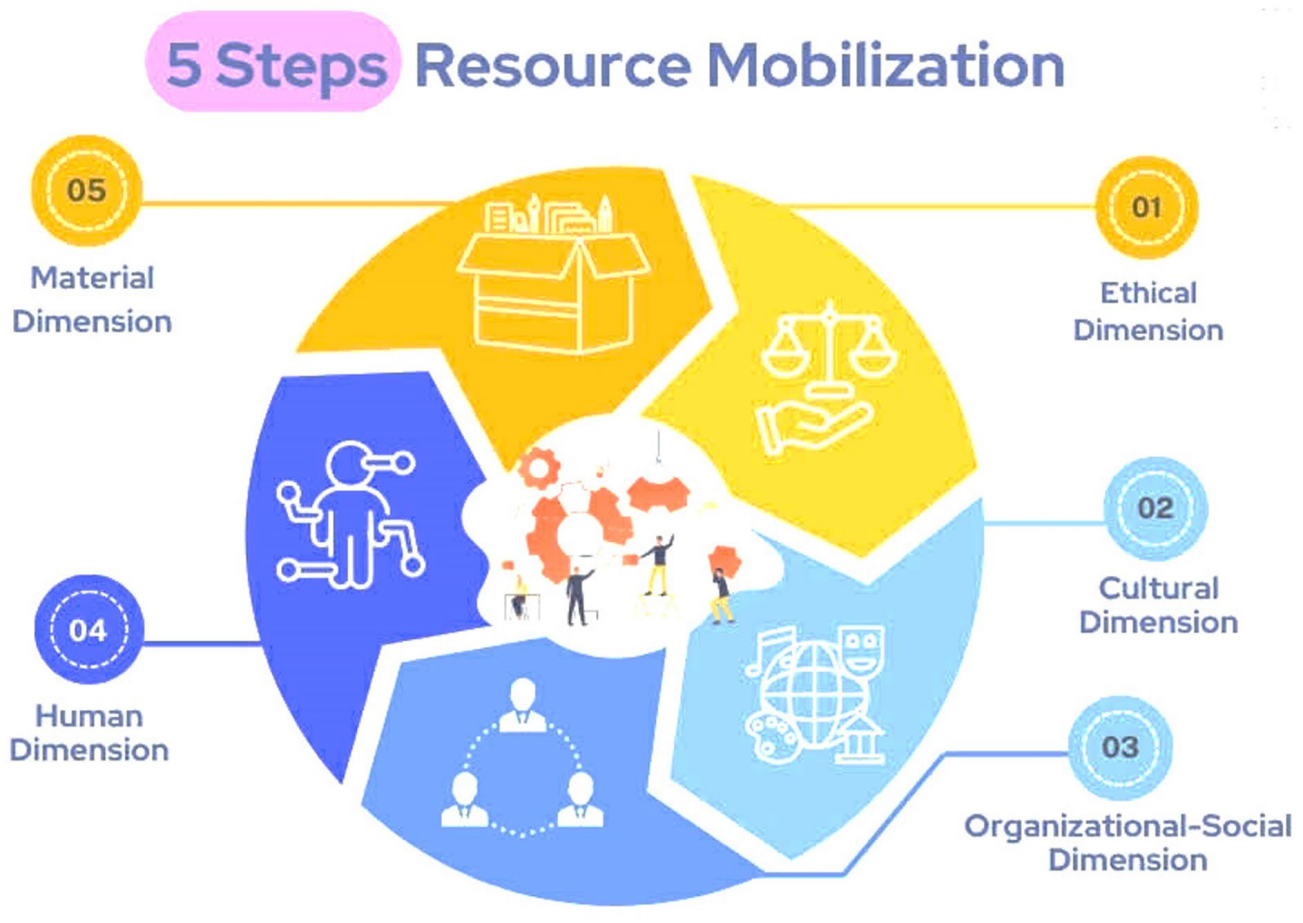
Resource mobilization five key dimensions.

In response to Research Question 2, the Khanom movement demonstrated a multidimensional strategy of resource mobilization, conceptualized in five interrelated dimensions: ethical, cultural, organizational-social, human, and material (Figure 2). Figure 2 is an analytically constructed framework developed by the author to synthesize empirical patterns observed in the Khanom case. It does not propose a causal model but serves as a heuristic device to organize different forms of resources mobilized through collective action. The framework is informed by social movement scholarship, particularly resource mobilization and framing theories, which conceptualize collective action as dependent on moral commitment, cultural legitimacy, organizational capacity, human skills, and material support (Della Porta, 2020b). The five dimensions were refined inductively through iterative coding and constant comparison across interviews, observations, and documentary materials.

Ethically, the movement cultivated a shared moral responsibility to protect community well-being and future generations. Culturally, it mobilized local ecological knowledge and traditional livelihoods as sources of legitimacy. Organizationally, informal networks and community associations facilitated coordination and information dissemination. Human resources particularly university-educated youth were critical in conducting research, engaging media, and articulating policy critiques. Material resources were sustained through community fundraising and in-kind support. As one resident activist explained:

*“Mobilization empowers the community to resist and negotiate with government agencies, providing us with motivation and a clear purpose to protect our home.” (P01).*

Over time, symbolic actions such as banners and signage evolved into sustained advocacy incorporating scientific evidence, legal challenges, and engagement with broader civil society networks. Environmental and public health concerns including perceived cancer risks associated with existing power plants further reinforced collective commitment.

#### 5.3.3 Governance tensions and developmental implications

Addressing Research Question 3, the analysis highlights how discursive resistance reshaped local governance dynamics, even without directly determining policy outcomes. Residents consistently criticized EGAT and state agencies for procedural deficiencies, particularly the absence of comprehensive Environmental Impact Assessments and meaningful consultation. These failures eroded institutional legitimacy and reinforced perceptions of exclusionary governance.

While the eventual suspension of nuclear power development in Khanom cannot be attributed solely to local activism, the movement significantly altered public narratives and expanded the political space for alternative development imaginaries. By articulating sustainability as ecological resilience, social justice, and community agency, the movement challenged the hegemony of growth-centric planning. As a local vendor reflected on the profound existential threat posed by the project:

*“When they talk about development, they always show us charts of money, jobs, and the ‘national interest.’ But for us in Khanom, development is not a spreadsheet; it is the air we breathe and the sea that feeds our grandchildren. I remember my father saying that this land doesn’t belong to us we are just borrowing it from those who come after us. If the nuclear plant comes, that sacred bond is broken. It’s not just about the fear of an accident; it’s about the feeling that our way of life is being treated as disposable. Development is not just about money. It’s about keeping our land and our children safe, and about the right to say, ‘this is our home, and we decide its future.’ If we lose the sea, we lose our soul, and no amount of compensation can buy that back.” (P01).*

This reframing underscores the role of grassroots resistance in transforming development from a technocratic project into a contested moral and political terrain, with enduring implications for Thailand’s environmental governance. The resistance in Khanom demonstrated that when institutional trust is absent, technical justifications from state agencies like EGAT fail to gain traction, regardless of their scientific complexity.

### 5.4 Knowledge brokers and intermediary actors in community resistance

Beyond grassroots mobilization and local leadership, the Khanom resistance movement was significantly shaped by the presence of intermediary actors, particularly local university graduates and students who acted as knowledge brokers between the community, civil society organizations, and external expert networks. These actors played a critical role in translating technical, legal, and environmental knowledge into locally meaningful narratives, thereby strengthening the movement’s discursive capacity. Interview data indicate that students and young graduates were frequently responsible for interpreting Environmental Impact Assessment (EIA) documents, summarizing scientific information on nuclear risk, and reframing such knowledge in accessible language during community meetings. One participant described this role as:

*“Helping people understand what the documents really meant for everyday life, not just what the experts said on paper.” (P07).*

Rather than positioning themselves as external experts, these intermediaries emphasized their identity as community members with shared histories and obligations. As one student explained:

*“I didn’t speak as a specialist. I spoke as someone whose family fishes here, just like everyone else.” (P12).*

This positionality enhanced trust and facilitated the circulation of knowledge across generational and social boundaries. In this sense, knowledge brokerage in Khanom was not merely informational but relational. Students operated at the intersection of formal education and lived experience, enabling the movement to engage with state rationalities of “technical safety” while simultaneously articulating alternative visions of sustainability grounded in local livelihoods, coastal ecologies, and moral responsibility to future generations. Through this process, expert discourse was not rejected outright, but selectively translated and re-embedded within community-based moral frameworks.

Importantly, the findings reveal a significant variation in how intermediaries operated within the movement, moving beyond a monolithic view of knowledge brokerage. Some students and university graduates primarily functioned as technical translators, focusing on deconstructing complex scientific data, legal language in EIA reports, and bureaucratic procedural requirements into accessible local dialects. In contrast, others acted as relational facilitators, whose primary role was not just to inform, but to bridge the social gaps within the community. They focused on organizing safe spaces for dialog, mediating intergenerational tensions between traditional leaders and younger activists, and providing emotional support to hesitant community members. A local elder (P19) reflected on the profound impact of this relational approach:

*“In the past, when officials came, we felt small and ignorant because we didn’t understand their technical terms. But the young people—our children who came back from university they changed that. They didn’t just come to lecture us; they helped calm our fears and explain things in a way that respected our life by the sea. They listened to our worries first. That made people less afraid to speak up in public meetings. It wasn’t just about the ‘science’ of nuclear power; it was about feeling that our voices finally had a place in the conversation.” (P19).*

This diversity of roles underscores that knowledge brokerage was not a uniform activity but a flexible and context-dependent practice. It illustrates how epistemic authority in Khanom was built not only through “knowing the facts” but through the “pedagogy of listening” and social embeddedness, which allowed the movement to maintain internal cohesion despite external pressures. From an analytical perspective, these intermediary practices can be understood as a form of discursive power. By translating expert knowledge into locally resonant terms, student intermediaries challenged the state’s monopoly over legitimate risk narratives without directly confronting institutional authority. Power, in this sense, operated through the ability to define what counted as credible knowledge and whose interpretations were considered legitimate in public discussion. Knowledge brokerage thus functioned as a mechanism through which communities reclaimed epistemic authority within a highly centralized energy governance system.

The role of student intermediaries in Khanom resonates with findings from studies of energy-related social movements in China and Singapore, where educated youth and professionals often function as mediators between state-led development agendas and community concerns. In China, for instance, environmental protests against nuclear and waste-to-energy projects have highlighted the importance of technically literate actors who translate risk knowledge into culturally resonant claims, enabling communities to challenge expert-driven state narratives without directly confronting political authority. Similarly, research on environmental governance in Singapore points to the emergence of policy-savvy intermediaries who mobilize scientific discourse and legal frameworks to negotiate limited spaces for civic participation within a highly centralized political. However, unlike these cases where intermediary actors often operate within constrained or elite policy circles the Khanom case demonstrates a more embedded and grassroots-oriented form of knowledge brokerage.

In Khanom, students did not primarily seek institutional recognition or policy reform through formal channels. Instead, their brokerage was oriented toward collective empowerment: strengthening community confidence, legitimizing local knowledge, and reinforcing resistance as a morally grounded and socially inclusive process. This variation suggests that while knowledge intermediaries are a common feature of energy conflicts across Asia, their functions and political effects are shaped by local histories of activism, social capital, and state–citizen relations.

The Khanom case extends existing literature by illustrating how knowledge brokerage can function as a form of resource mobilization that is simultaneously cognitive, social, and ethical. Rather than treating students as a homogeneous category of “educated activists,” the findings highlight variation in intermediary roles from technical translators to relational facilitators within a single movement. This insight contributes to broader debates on social movements and sustainable development by demonstrating that resistance to large-scale energy infrastructure is not solely driven by material grievances or ideological opposition, but also by the capacity of movements to mediate knowledge across social divides. In this regard, Khanom offers an alternative model of intermediary politics in Southeast Asia, one rooted in community embeddedness rather than technocratic advocacy.

## 6 Policy implications for sustainable energy governance in Thailand

Based on the findings of this study, the following policy recommendations are proposed to enhance the legitimacy and inclusivity of energy governance in Thailand:

- Institutional Reform for Meaningful Participation: Energy regulatory bodies must transition from top-down information dissemination to a “deliberative governance” model that grants local communities legally binding roles in the decision-making process for large-scale infrastructure projects.
- Ensuring Independent and Transparent Impact Assessments: The Environmental and Health Impact Assessment (EHIA) process should be conducted by independent third-party agencies, separate from state-led developers like EGAT, to ensure scientific neutrality and restore public trust in environmental data.
- Integration of Community-Led Energy Alternatives: National energy plans should formally recognize and subsidize decentralized, small-scale renewable energy initiatives managed by local cooperatives, moving beyond the current focus on centralized, high-risk energy sources.
- Adoption of a “Social Justice” Framework in Policy: Energy policy must move beyond technocratic efficiency and prioritize the protection of local livelihoods and cultural identities, ensuring that the “burden of development” does not disproportionately fall on marginalized coastal and agricultural communities.
- Establishment of Independent Conflict Mediation Mechanisms: To mitigate deep-seated distrust, the government should establish an autonomous energy ombudsman or a neutral mediation platform to facilitate transparent dialog between state agencies and resistance movements before project approvals.

## 7 Discussion

This study uses the Khanom nuclear power plant conflict to advance sociological debates on community resistance, knowledge, and sustainable development within contested energy governance. Rather than treating resistance as a reactive response to state-led infrastructure, the findings demonstrate how resistance operates as a productive social process through which meanings of development, sustainability, and authority are actively reconfigured. The contribution of the study thus lies not in documenting opposition to nuclear power per se, but in theorizing how development conflicts function as sites of discursive struggle and moral contestation. By integrating insights from Foucauldian discourse analysis, social movement theory, and critical development studies, the Khanom case illustrates how power operates through knowledge, legitimacy, and meaning-making, rather than through coercion alone (Foucault, 1977; Escobar, 1995).

The first contribution concerns the reconceptualization of community resistance as a process of discursive reconfiguration. The Khanom movement did not merely reject a nuclear power plant; it challenged a dominant development narrative that equates large-scale energy infrastructure with progress, national security, and modernization. While global mitigation discourses increasingly frame nuclear energy as a necessary instrument for decarbonization (Intergovernmental Panel on Climate Change, 2022), the Khanom case reveals how such technocratic justifications often overlook local socio-ecological conditions and lived vulnerabilities. Community actors redefined development in terms of environmental safety, livelihood continuity, food security, and intergenerational responsibility. This finding extends scholarship on discursive politics and environmental conflict by demonstrating that resistance is not only oppositional but generative, producing alternative development imaginaries rooted in everyday experience (Hajer, 1995; Dryzek, 2013).

The second contribution lies in identifying the role of locally embedded university graduates as discursive intermediaries. These actors translated scientific, legal, and policy knowledge into narratives that resonated with local concerns and moral frameworks. This brokerage process is critical because, as the International Atomic Energy Agency emphasizes, stakeholder involvement is a formal pillar of nuclear governance (International Atomic Energy Agency, 2007). The Khanom experience demonstrates, however, that institutional participation mechanisms alone are insufficient to generate legitimacy without intermediaries who possess social capital, shared identity, and accountability to place. Epistemic authority in this context is not simply derived from technical expertise but is socially produced through trust and relational embeddedness. This finding contributes to sociological debates on knowledge and power by showing how expertise circulates horizontally within communities, rather than flowing unidirectionally from experts to lay publics.

The third contribution concerns the reconceptualization of sustainability as a contested moral discourse. While policy-oriented approaches often treat sustainability as a technical objective to be optimized through expert planning, the Khanom case reveals sustainability as a normative field structured by competing moral claims. State and corporate actors mobilized sustainability through technocratic language emphasizing energy security and national development. In contrast, community actors articulated sustainability as a moral commitment to health, dignity, ecological integrity, and the continuity of everyday life. This supports broader critiques in political ecology and energy democracy scholarship that sustainability is not a neutral concept but a site of political struggle, where global scientific consensus must be negotiated against the lived realities of communities at the frontlines of energy transitions.

Taken together, the Khanom case advances sociological theory by integrating discourse, knowledge, and collective action into a unified analytical framework. By conceptualizing resistance as discursive reconfiguration, highlighting the role of intermediary actors, and reframing sustainability as a moral discourse, the study demonstrates how development conflicts function as dynamic arenas in which alternative futures are actively imagined and negotiated. These contributions directly address the study’s research questions by showing how lived experience is translated into discursive practice and how such practices contest technocratic authority within centralized governance regimes. In comparative perspective, the Khanom case shares similarities with anti-nuclear movements in India, Taiwan, and South Korea, particularly in its emphasis on risk, democratic deficits, and technocratic dominance. However, it also reflects context-specific dynamics shaped by Thailand’s centralized energy governance, the institutional authority of EGAT, and constrained participatory mechanisms under semi-authoritarian conditions. These features help explain why resistance in Khanom relied heavily on discursive strategies and intermediary actors rather than sustained mass mobilization, contributing a Southeast Asian perspective to comparative scholarship on nuclear politics.

Finally, the findings remain highly relevant beyond the immediate temporal scope of the study. The discursive struggle that intensified between 2016 and 2023 reflects broader patterns in Thai environmental governance, where state reliance on technical-led sustainability narratives continues to prompt the emergence of counter-discourses grounded in lived experience. As of 2026, these dynamics persist, underscoring the enduring significance of discursive contestation in shaping the legitimacy of energy transitions in the Global South.

## 8 Conclusion

This study examined how community resistance to the proposed nuclear power plant in Khanom emerged, operated, and reshaped development discourse through locally grounded yet politically consequential forms of collective action. In response to Research Question 1, the findings demonstrate that resistance in Khanom took the form of a discursive reconfiguration of development, whereby local actors challenged state-led narratives of progress and articulated alternative meanings of sustainability rooted in environmental safety, livelihood security, and intergenerational responsibility.

Addressing Research Question 2, the analysis highlights the critical role of locally embedded university graduates as discursive intermediaries who translated technical and scientific knowledge into culturally resonant narratives. Through this process, they mobilized collective action, produced localized epistemic authority, and enabled community members to contest expert-driven energy governance on their own terms. With regard to Research Question 3, the study shows that these discursive and organizational practices exposed enduring structural tensions between centralized energy governance and local participatory aspirations, revealing sustainability not as a neutral policy objective but as a contested moral and political discourse.

Situated within a broader historical perspective, the Khanom case reflects a contemporary manifestation of global green ideas and movements that have evolved since the nineteenth century through recurring critiques of industrial modernity, technocratic authority, and environmentally destructive development trajectories. While differing from early conservationist or post-materialist environmental movements in the Global North, resistance in Khanom resonates with a longer genealogy of green thought by foregrounding moral claims about nature, risk, and responsibility for future generations. In this sense, the case illustrates how globally circulating green ideas are re-articulated through local histories, social relations, and livelihood concerns in the Global South.

Taken together, the findings underscore the importance of understanding development conflicts as sites of discursive struggle in which meanings of sustainability, progress, and collective futures are actively negotiated. Beyond Thailand, the analytical framework advanced in this study contributes to broader debates on community resistance, knowledge brokerage, and the evolving politics of green movements, demonstrating how global environmental ideas gain political traction through locally embedded struggles over development and energy governance.

## Data Availability

The original contributions presented in the study are included in the article/supplementary material, further inquiries can be directed to the corresponding author.
